# Utilizing a Structural Mechanics Approach to Assess the Primary Effects of Injury Loads Onto the Axon and Its Components

**DOI:** 10.3389/fneur.2018.00643

**Published:** 2018-08-06

**Authors:** Annaclaudia Montanino, Svein Kleiven

**Affiliations:** Division of Neuronic Engineering, Royal Institute of Technology (KTH), Huddinge, Sweden

**Keywords:** diffuse axonal injury, axon, axon finite element model, axolemma, microtubules

## Abstract

Diffuse axonal injury (DAI) occurs as a result of the transmission of rapid dynamic loads from the head to the brain and specifically to its neurons. Despite being one of the most common and most deleterious types of traumatic brain injury (TBI), the inherent cell injury mechanism is yet to be understood. Experimental observations have led to the formulation of different hypotheses, such as mechanoporation of the axolemma and microtubules (MTs) breakage. With the goal of singling out the mechanical aspect of DAI and to resolve the ambiguity behind its injury mechanism, a composite finite element (FE) model of a representative volume of an axon was developed. Once calibrated and validated against published experimental data, the axonal model was used to simulate injury scenarios. The resulting strain distributions along its components were then studied in dependence of strain rate and of typical modeling choices such as the applied MT constraints and tau proteins failure. Results show that oversimplifying the MT bundle kinematic entails a systematic attenuation (*cf* = 2.33) of the computed maximum MT strain. Nevertheless, altogether, results support the hypothesis of axolemma mechanoporation as a cell-injury trigger. Moreover, for the first time the interconnection between the axolemma and the MT bundle is shown to play a role in damage localization. The proposed FE axonal model is a valuable tool to understand the axonal injury mechanism from a mechanical perspective and could be used in turn for the definition of well-informed injury criteria at the tissue and organ level.

## 1. Introduction

Traumatic brain injury (TBI) is defined as an insult to the brain from a mechanical force, which leads to a temporary to permanent impairment of brain function. TBI today is regarded as a major social and economic burden for society (1). In the world, in fact, 10 million people are estimated to sustain TBIs every year and the total estimated cost is of 76.5 billion dollars in the United States (2) and 33 billion euros in Europe (3). Despite TBI pathophysiology being complex and heterogeneous, diffuse axonal injury (DAI)—one of close-head TBI pathological evidences—was recognized as the main mechanism of injury in 40−50% of the hospitalized cases of TBI in the United States (4). At the histopathological examination, DAI appears as a multifocal damage of the white matter axons (5), which have hence been regarded as neurons' most vulnerable compartment. To prevent the occurrence of DAI, for example by developing effective protective systems, the mechanism of injury must first be uncovered. So far, injury thresholds have been proposed in the literature both at the organ and tissue level using finite element (FE) models (6–9). Nonetheless, a full understanding of DAI cannot prescind investigations at the cellular scale, where structural damage and functional injury coexist.

In the last decade researchers have addressed this problem experimentally. Neurons have been tested mechanically not only to capture their mechanical properties (10–14), but also to reveal their behavior in injurious scenarios, namely by the application of dynamic loads onto the cells (15–18). Experimental observations have led to the formulation of different hypotheses regarding the cellular injury mechanism. While some have postulated a membrane damage-driven cell injury mechanism (also referred to as axolemma mechanoporation) (19–22), other studies have supported, instead, microtubules disruption as a cell injury trigger (23). The latter has also been corroborated by the interesting co-localization of microtubule failure and tau protein accumulation sites and axonal swellings (24). Among the observed axonal alterations, neurofilaments compaction (25–27), and microtubule associated protein (MAP) tau failure have also been highlighted and found to be correlated with neurodegenerative diseases such as Alzheimer's and dementia (5).

More recently, researchers have tried to explain a mechanism for cell injury by means of numerical and analytical models. Most of these efforts have, however, targeted the microtubules bundle rather than the axon as a whole. Discrete bead-spring models have been used to investigate the mechanical behavior of this axonal substructure under different types of loads (28–30). For the same structure, a modified shear-lag model was proposed by Ahmadzadeh et al. (31, 32) to quantify the loads experienced by different bundle components. More recently a finite element model of the microtubule bundle consisting of dynamically changing microtubules and crosslinks was proposed (33). It is however fundamental to notice that in these studies inferences on the whole axonal failure are based on observations made on the sole bundle response. Moreover, while modeling complex microtubules and crosslinks behavior, these studies allow only for axial displacements and deformations of the microtubules. As a result of this constraint, the bundle kinematics, which was shown to consist instead of an initial MTs bend-dominated phase followed by a stretch-dominated phase (28), is simplified. Nevertheless, the influence of a realistic bundle kinematic on axonal injury metrics is yet to be quantitatively assessed.

It appears evident that the composite nature and the non-affine behavior of the axonal cytoskeleton play an important role in the injury mechanism (34, 35). However, to date, numerical studies have not considered the axon in its entirety, but only compartments of it. In fact, although a simplified FE model of the whole axon was proposed by Zhu et al. (36), this was only used to derive effective spring stiffnesses for consecutive axonal tracts in a static regime. The axon was then represented as a series of springs and used as such to perform dynamic simulations. This representation, however, does not provide potentially relevant local information such as the deformation of different axonal compartments.

The aim of this study is to quantitatively address the existent hypotheses in a comprehensive way. To understand whether mechanoporation of the axolemma or MTs failure could be considered an axonal injury initiating mechanism, an FE model of the axon was developed for this study. The model, which includes all the mechanically relevant substructures, once calibrated and validated against experimental data, was first used to assess the influence of bundle kinematics on axonal failure metrics (maximum MTs and axolemma strain and axolemmal thinning). The same axonal failure metrics were then observed to assess their dependence on strain rate and tau protein failure, the objective being to answer the question “what is the axonal injury mechanism?” from a structural mechanics perspective.

## 2. Materials and methods

### 2.1. Axonal FE model

**Figure 1:**
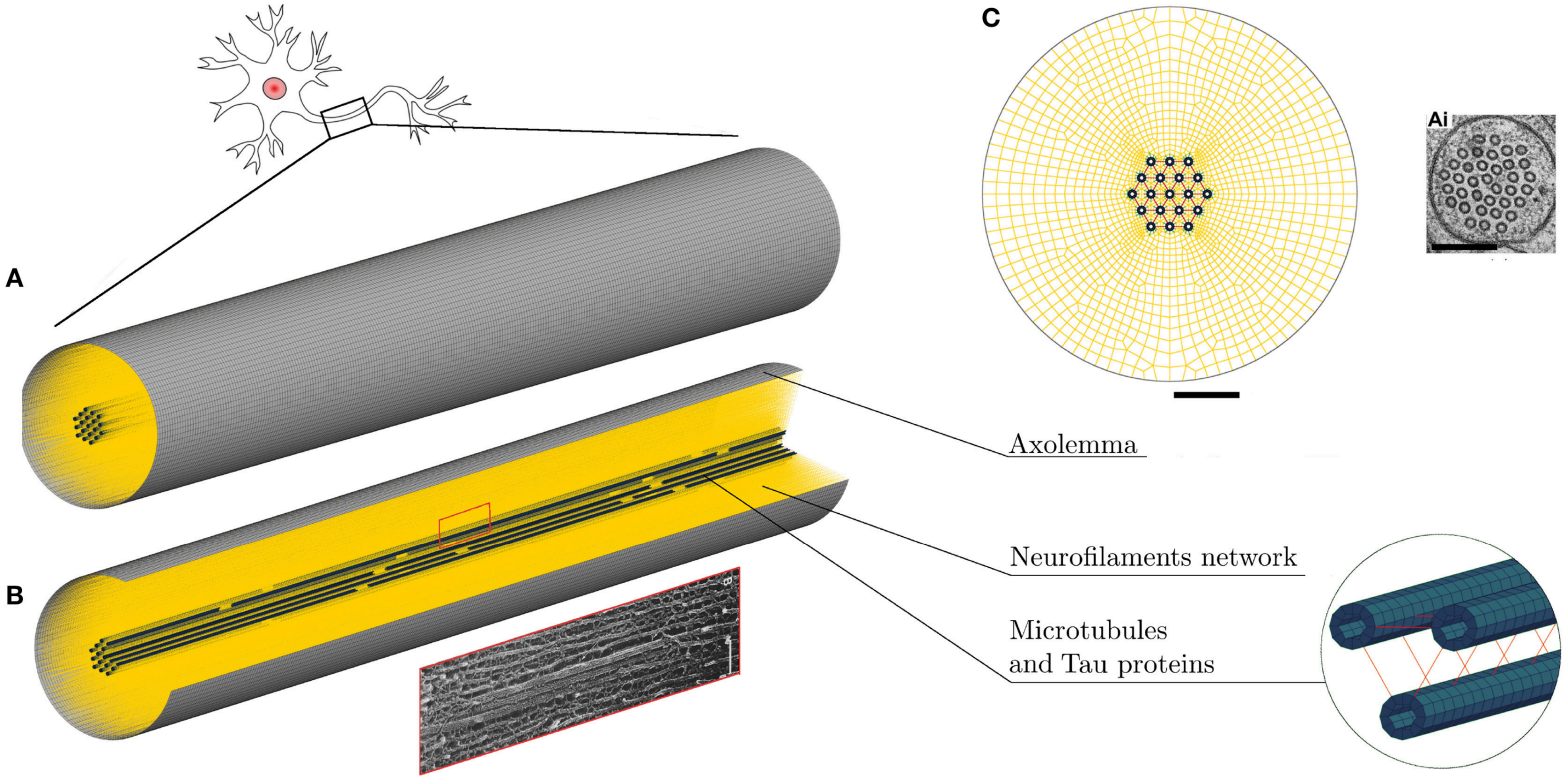
**(A)** FE axonal model. **(B)** A quarter of the model was removed to ease the visualization of its sub-structures: axolemma as a shell layer, MTs (blue hollow solid elements cylinders) cross-linked by tau proteins (red) and the beam-meshed NFs network (yellow). In the rectangle below, a micrograph (41) shows a detail of the axonal cytoskeleton with three MTs surrounded by parallel NFs. **(C)** Front view of the model that shows its mesh symmetry and the MTs arrangement in comparison with an electron micrographs of *C. elegans* touch receptor neuron (42) (scale bars 200 nm). Micrograph (41) reproduced with permission of ROCKEFELLER UNIVERSITY PRESS in the format journal/magazine via Copyright Clearance Center.

Based on the fact that axonal injury is a mechanically dominated problem, a finite element (FE) structural mechanics approach was chosen to investigate axonal behavior. Although the FE method cannot represent the entirety of the real problem physics, in this case it suits the purpose of singling out the mechanical aspects of a micrometric structure that could not otherwise be numerically investigated and to obtain an estimation of the loads sustained by different axonal components. The same approach has been used for example to model cells subjected to mechanical loads (37–39) and even nanometric structures such as membrane protein channels with FE-represented protein helices and loops of subnanometric thickness (40). Therefore, a FE model of a representative volume (RV) of the axon was developed in LS-DYNA ® (Figure 1). The model aims at representing the entirety of the mechanically relevant axonal substructures with the approximations that are necessary to withhold its generality. The axon RV was hence modeled as an 8 μm long- cylinder [length chosen as in (28) to contain a unit length of the microtubules bundle] with a diameter of 1.5 μm, as the average diameter of primary rat cortical neurons (14). The model, like the axon, is a composite structure that includes the following subcomponents: microtubule bundle, neurofilaments network, and axolemma-actin cortex complex.

Microtubules (MTs) in the axon have uniform orientation, with their plus ends toward the axon tip (43). Cross-linked by microtubule associated protein (MAP) tau, they form dense parallel arrays (bundles), which make a major contribution to axonal stiffness and structural integrity. Given the desired axonal radius and the MTs average cross-sectional densities (44–46), 19 rows of MTs were included with a distance of 30 nm between neighboring MTs rows (47) (Figure 1C). In this FE axonal model, as in all the previous studies focused on MTs bundle failure, all axonal MTs were grouped in a single central *MT bundle*, this representing the worst case scenario. The higher the number of MTs in a bundle (in the range of reported cross-sectional densities), the higher is the traction that these exert on one another. The MTs are represented as hollow cylindrical structures, of 14 nm-inner and 25 nm-outer diameter, made of linear elastic solid hexahedral elements [E = 830 MPa (48)]. Being 4.02 μm the average continuous length of axonal microtubules (49), in order to preserve the periodicity of the structure, two discontinuities of 0.15 μm were included in each of the 19 rows. The MTs have been finally cross-linked using discrete beam elements representing tau proteins (Figure 1B). The cross-connections between MTs were reported to be less numerous than the ones between neurofilaments (30–50 nm) (50), hence, from visual inspection, tau proteins spacing was set to be constant and equal to 120 nm. Moreover, in a previous sensitivity study (28), it was shown that, while cross-link density mainly affects the initial MTs bending energy (<7% difference in MTs bending energy between bundles with 75 and 100 nm cross-links spacing), it does not affect their elastic stretching energy. In our model these elements were assigned linear viscoelastic properties derived from Ahmadzadeh et al. (31, 32) with a further simplification, namely not considering bonding/debonding probability. The effects on the global axonal response of a tensile strain failure threshold for these proteins was also studied.

The axonal membrane, or *axolemma*, is a lipid bilayer structure that separates the intracellular environment from the extracellular one, whose protein channels regulate the electrical activity of the neuron. The axonal membrane sits on a periodic actin-spectrin skeleton (51), which has been shown to greatly contribute to the membrane stiffness (52). In the FE axonal model, the axolemma and the underlying actin-spectrin cortex were treated as a unique structure, which was modeled as a layer of fully integrated, 200 nm-thick shell elements that were assigned linear viscoelastic behavior (37).

The third and last axonal component to be modeled, is the *neurofilaments (NFs) network*. NFs are the axonal specific intermediate filaments that run with their backbones parallel to the MTs array and are 10 times more numerous than the latter (53). These filaments form a lattice that fills up the space and their projection domains (or side arms) are thought to determine the axonal diameter (54), maintain the integrity of the NFs network and anchor it to the plasma membrane and the MTs (55). These side arms have been reported to have a 20 to 30–50 nm spacing and an average length of 23 nm (56–58). Based on this information, the NFs network was modeled as a dense mesh of horizontal beams with radially departing beams (23 nm long on an average and with spacing of 30nm in the axial direction) mimicking respectively the arrangement of backbones and projection domains (Figure 1C). Few studies have measured the bulk properties of NFs networks *in vitro* and revealed their viscoelastic behavior (59–62). However, it is currently not clear how these relate to the *in vivo* properties. The *in vivo* highly organized arrangement of NFs, as well as the cross-linking system, differs from the one resulting from an artificial preparation. Therefore, the global elasticity of this axonal component was derived through model calibration. Finally, the few elements linking NFs and MTs were modeled as discrete beams with an average length of 7 nm and spacing of 30 nm (63) and (57). These elements, which are an idealization of dynein or kinesin motor proteins were assigned a spring stiffness k = 1 μN/m, in the same order of magnitude as the one for kinesin stiffness (64). A summary of the model components mechanical properties can be found in Table 1.

**Table 1:** Model material parameters.

**Axonal component**	**Element type**	**Material**	**References**
Microtubules	Solid	Linear elastic	(48)
		*E* = 830*MPa*	
		ν = 0.37	
Axolemma	Shell	Linear viscoelastic	(37)
		$G_{0}=3.333\cdot 10^{-4}MPa$	
		$G_{\infty }=1.333\cdot 10^{-5}MPa$	
		τ = 3, 000*s*	
		ν = 0.499	
Tau proteins	Discrete beam	Linear viscoelastic	(31, 32)
		*K* = 5·10^−5^*N*/*m*	
		μ = 2.205·10^−4^*Ns*/*m*	
NFs elements	Discrete beam	Linear viscoelastic	calibr.
		*K* = 7.5·10^−5^*N*/*m*	
		μ = 1·10^−7^*Ns*/*m*	
MTs-NFs links	Discrete beam	Linear viscoelastic	(64)
		*K* = 1·10^−6^*N*/*m*	
		μ = 1·10^−7^*Ns*/*m*	

### 2.2. Model calibration and validation

**Figure 2:**
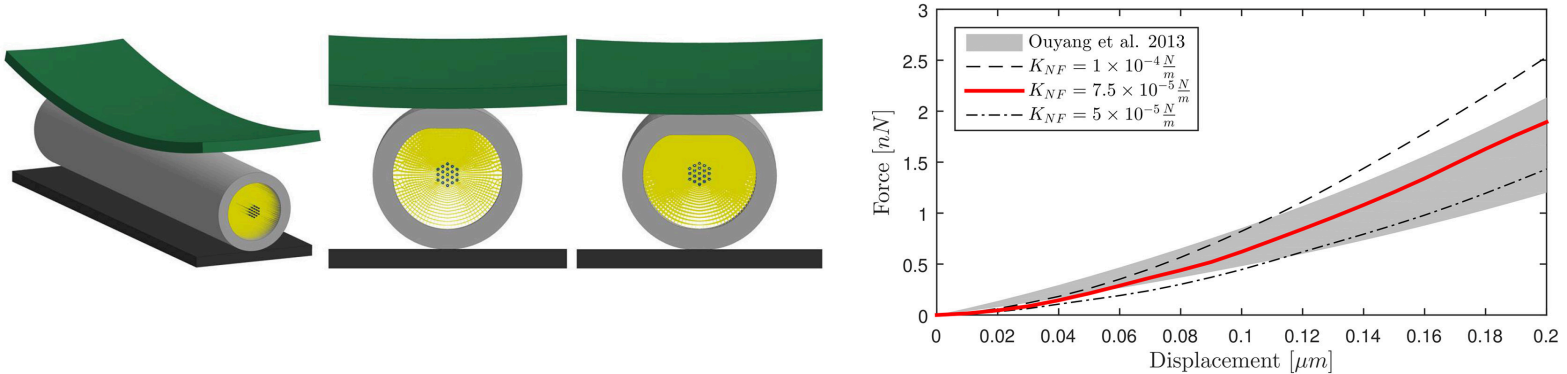
Numerical compression setup **(left)**. Shell thickness is also shown here. Axonal compression results **(right)**. The numerical results for three different NFs network stiffnesses are here compared against experimental results as a gray shaded area (65).

The FE axonal model was first calibrated against axonal compression data (65) to obtain an appropriate elasticity for the NFs network. A polystyrene bead (E = 3 GPa, ν = 0.34) of radius 12.5 μm was modeled with shell elements right above the axon, which was placed on a rigid flat surface (Figure 2, left). The bead was then prescribed a downward displacement at 4.08 μ*ms*^−1^ speed until the displacement reached the 10% of the axon height to avoid including the substrate stiffness onto the measurement (66). The resulting bead-axon contact forces were then compared against the corresponding data (65) (Figure 2, right) multiplied by a factor $f={\left(\frac{r_{mod}}{r_{exp}}\right)}^{\frac{1}{4}}=1.10$ derived from Formula 6 in Ouyang et al. (65) to account for the difference between the axonal model radius and the experimental one (*r*_*mod*_ = 1.5*r*_*exp*_). As a result of this calibration, the chosen NFs element stiffness was $K_{NF}=7.5\times 10^{-5}\frac{N}{m}$. Since rheological compression experiments have not been performed on axons yet, the viscosity of the NFs beam elements was arbitrarily set with a viscosity coefficient 10^−7^*N*·*s*/*m* that was checked not to substantially affect the results.

**Figure 3:**
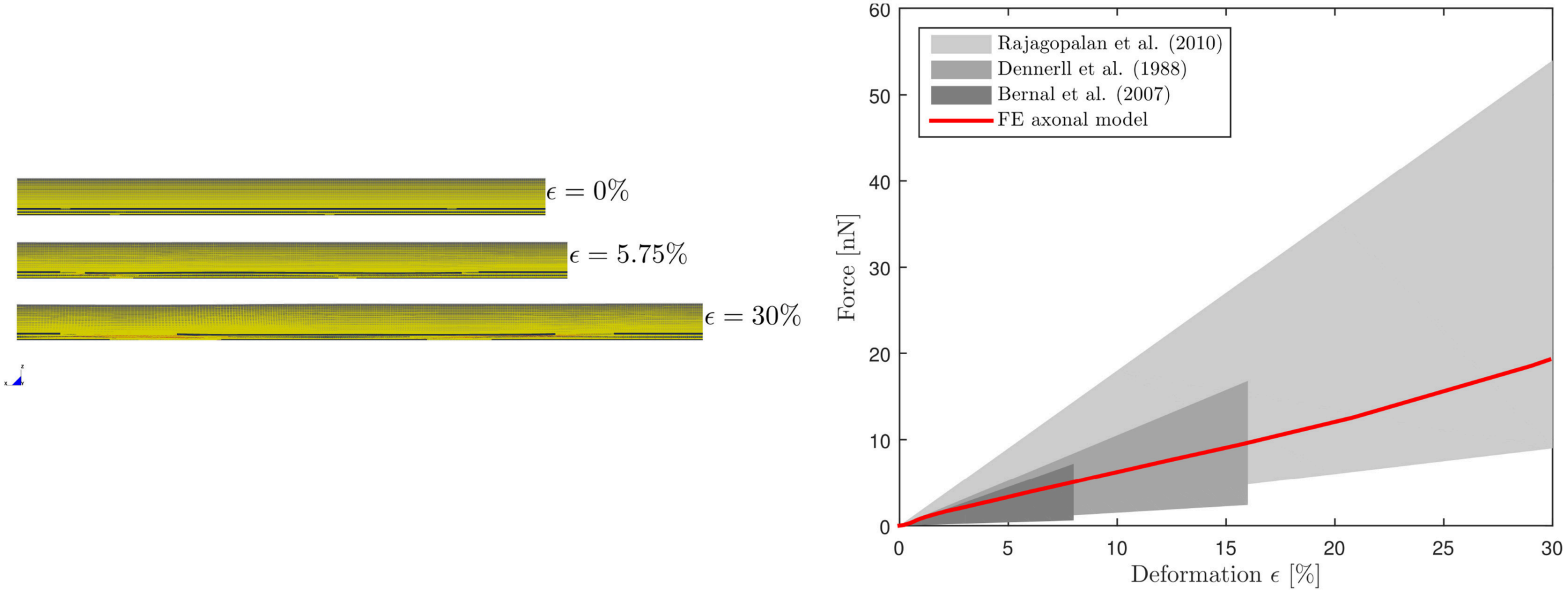
Validation of the FE axonal model in tension. Simulation setup **(left)**. On the **(right)**, numerically-derived Force-Deformation curve (*red*) is plotted against literature data from three different studies (gray shaded areas).

The calibrated model was then validated in tension (Figure 3, left). Symmetry conditions were enforced on a quarter model to reduce the computational expense. The left face of the model was constrained in the axial direction but was free to contract in the other two directions. A displacement was applied on the right face to reach ε = 30% global strain with $\dot{\varepsilon }=0.17s^{-1}$ strain rate, correspondent to the speed v(t)=25 μ*ms*^−1^ applied in Bernal et al. (11). The effective Young's modulus *E*_*eff*_ of the composite model was derived dividing the engineering stress at the left boundary by the global applied strain. The axonal model spring stiffness was then calculated as $K=\left(E_{eff}A\right)/\overset{-}{L}$, where *A* is the cross-sectional area and $\overset{-}{L}$ is the mean axonal length reported in literature experiments used as a reference for our validation (10–12). Validation results are shown in Figure 3 (right), which purposefully highlights the strain ranges at which axons were tested in the reference studies. The FE model average spring stiffness (0.4 mN/m) is in good agreement with the experimentally derived ones [0.3 mN/m (10), 0.27 mN/m(11), and 0.5 mN/m (12)] up to 30% global strain.

### 2.3. Injury simulations

Tensile elongation of the axons has been shown to correlate with axonal injury. Based on experiments, cell-level injury thresholds have been proposed. These thresholds range from 5−10% (67, 68) to 18−21% (69, 70). To investigate axonal behavior at large strains, a family of 10 models was produced by slightly altering the baseline model, namely by moving the discontinuities locations along the rows, ensuring that the average MTs length within each model was kept equal to 4.02 μm as in the baseline model. Symmetric boundary conditions were enforced on each model, which was then numerically stretched up to a global strain ϵ_*axon*_ = 30% as previously described for the tensile validation. All the simulations were performed in LS-DYNA ® using implicit nonlinear dynamic solvers. The choice of using implicit dynamic solvers was dictated by the small dimension of the elements that makes it unfeasible to work in an explicit framework. The dynamic nature of the analysis, however, ensures that strain rate dependent phenomena can indeed be captured.

First, the effect of considering a realistic bundle kinematic on maximum compartmental strains was studied for strain rates ranging from the quasi-static to the low-dynamic regime ($\dot{\varepsilon }=0.001s^{-1}-1s^{-1}$). The resulting maximum von Mises (VM) strains in the MTs and in the axolemma were extracted in the two following configurations: when the MTs were allowed 6 degrees of freedom [free MTs, or realistic bundle behavior (28)] and when, instead, the MTs were allowed only 1 axial degree of freedom (constrained MTs) as proposed in Ahmadzadeh et al. (32) and de Rooij and Kuhl (33), the goal being the assessment of the effect that MTs bending has on the computed maximum MTs strain.

Given the influence of strain rate on axonal mechanics (71, 72), it was deemed important to assess the effect of this not only on the MT bundle, but also on the axolemma when these two compartments are connected. However, large strains and high strain rates irremediably increase the instability of the simulations hindering their convergence. Therefore, having previously assessed the effect of the MTs constraint, this was enforced for this part of the study and simulations were run for the whole family of models using the following strain rates: $\dot{\epsilon }=0.001,0.1,1,10,20,40s^{-1}$). To speculate upon the injury state of the axons in different scenarios, the following quantities were extracted: maximum von Mises (VM) strains in the MTs and axolemma compartments, as well as axolemmal thinning.

As cross-linkers of the MT bundle, tau proteins play an important role in load bearing capacity of the composite structure (31, 32). To study how the axonal response changes when these links fail, a strain failure threshold was introduced [at ϵ_τ_ = 100% (28)] and the same injury metrics were analyzed.

## 3. Results

### 3.1. Influence of bundle kinematics on injury metrics

**Figure 4:**
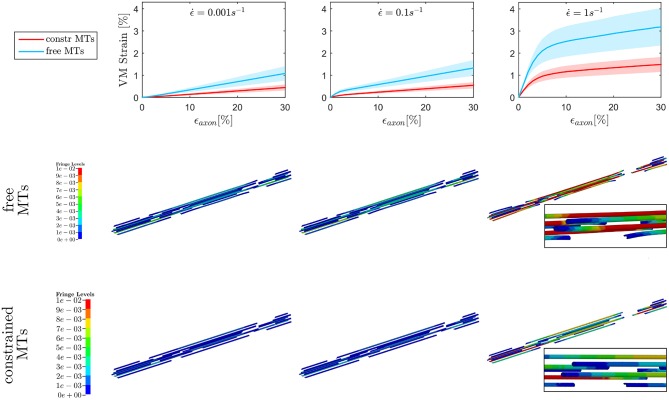
Maximum von Mises (VM) strain in the **MT bundle** (first row) computed when stretching the whole axon up to ε_*axon*_ = 30% at strain rates $\dot{\varepsilon }=0.001s^{-1}$, $\dot{\varepsilon }=0.1s^{-1}$, and $\dot{\varepsilon }=1s^{-1}$ from left to right. Plots show the results obtained considering a realistic bundle kinematic (free MTs) and a simplified one (constrained MTs). Solid line and shaded areas represent respectively the mean value and the standard deviation computed over a family of 10 models. On the second and third row, fringe plots for one of the models show the distribution of VM strains along the bundle at ε_*axon*_ = 30%, respectively in the free and constrained MTs configuration.

The first goal of this study was to assess the effect of MTs bending on injury indicators such as maximum MTs strain ϵ_*MTs, max*_ and maximum axolemmal strain ϵ_*Axol, max*_ when a simplified bundle kinematics is considered (constrained MTs) rather than a realistic one (free MTs). A total of 10 different models were tested with and without the aforementioned MTs constraint up to $\dot{\epsilon }=0.1s^{-1}$. However, only six models could be run till convergence at $\dot{\epsilon }=1s^{-1}$ in the free MTs condition, which stresses even more the need of quantifying this error to legitimately simplify the bundle kinematics. Figure 4, top row, shows the evolution of ϵ_*MTs, max*_ when the whole axon is stretched up to a global strain ε_*axon*_ = 30% at strain rates ranging from the quasi static to the low dynamic regime. First of all, it is apparent that, although the model family variance reaches the 8% at its maximum (for the maximum VM strain obtained at ϵ = 30% and $\dot{\epsilon }=1s^{-1}$ in the *free MTs* configuration), there is still a clear separation between the results obtained with the two configurations. For each strain rate, the results (maximum VM strains in the MT bundle) obtained with or without MT constraint were compared. The comparison was carried out using the two-sample *t*-test. Namely, for each time point, the *free MTs*-data were compared against the *constraint MTs*-data and resulted to consistently belong to populations with significantly different means (*p* <0.001). Moreover, from the second and third row of Figure 4, it can be observed that, as a result of MTs bending, the bundle packs around its axis and MTs experience sensibly higher VM strains. This difference was quantified at each strain rate for each model as the relative difference between results obtained with and without the constrained. This resulted to be on average *e* = 57 ± 1% and no trend was detected across strain rates. Fringe plots in Figure 4 complement the previous magnitude-focused information with a spatial-related one. At each strain rate, not only are the maximum strains reached in the free MTs configuration higher, but they also concern a higher number of elements along the MTs. Nevertheless, it can be stated that the element-wise maximum strain in the constrained scenario is a systematic fraction of that computed in the free MTs scenario.

**Figure 5:**
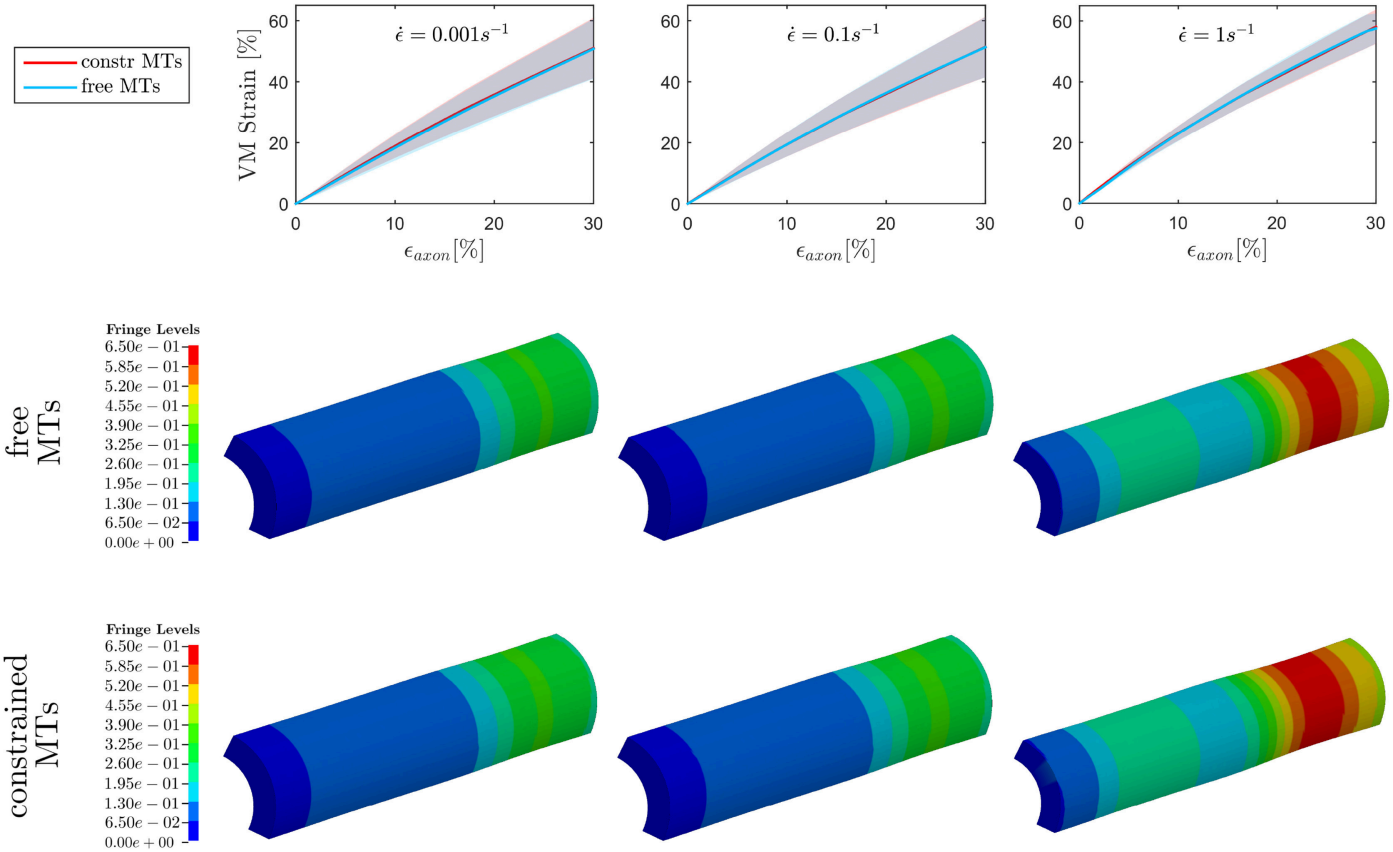
Maximum von Mises (VM) strain in the **axolemma** (first row) computed when stretching the whole axon up to ε_*axon*_ = 30% at strain rates $\dot{\varepsilon }=0.001s^{-1}$, $\dot{\varepsilon }=0.1s^{-1}$, and $\dot{\varepsilon }=1s^{-1}$ from left to right. Plots show the results obtained considering a realistic bundle kinematic (free MTs) and a simplified one (constrained MTs). Solid line and shaded areas represent respectively the mean value and the standard deviation computed over a family of 10 models. On the second and third row, fringe plots for one of the models show the strain distribution in the axolemma at ε_*axon*_ = 30%, respectively in the free and constrained MTs configuration.

On the other hand, when observing the results for ϵ_*Axol, max*_ (Figure 5), no difference could be detected between the constrained and unconstrained configuration, meaning that in our model the differences in bundle kinematics do not translate into differences at the axolemmal level. Figure 5, (first row), shows overlapping results between the constrained and free MTs conditions. It is worth noting from the fringe plots in Figure 5 (second, third row) the non homogeneous strain distribution on the axolemma. Despite being little dependent on the constraint enforced on the MTs, this strain concentration gets more evident with the increase of strain rate.

### 3.2. Strain rate effect on maximum compartmental strains

**Figure 6:**
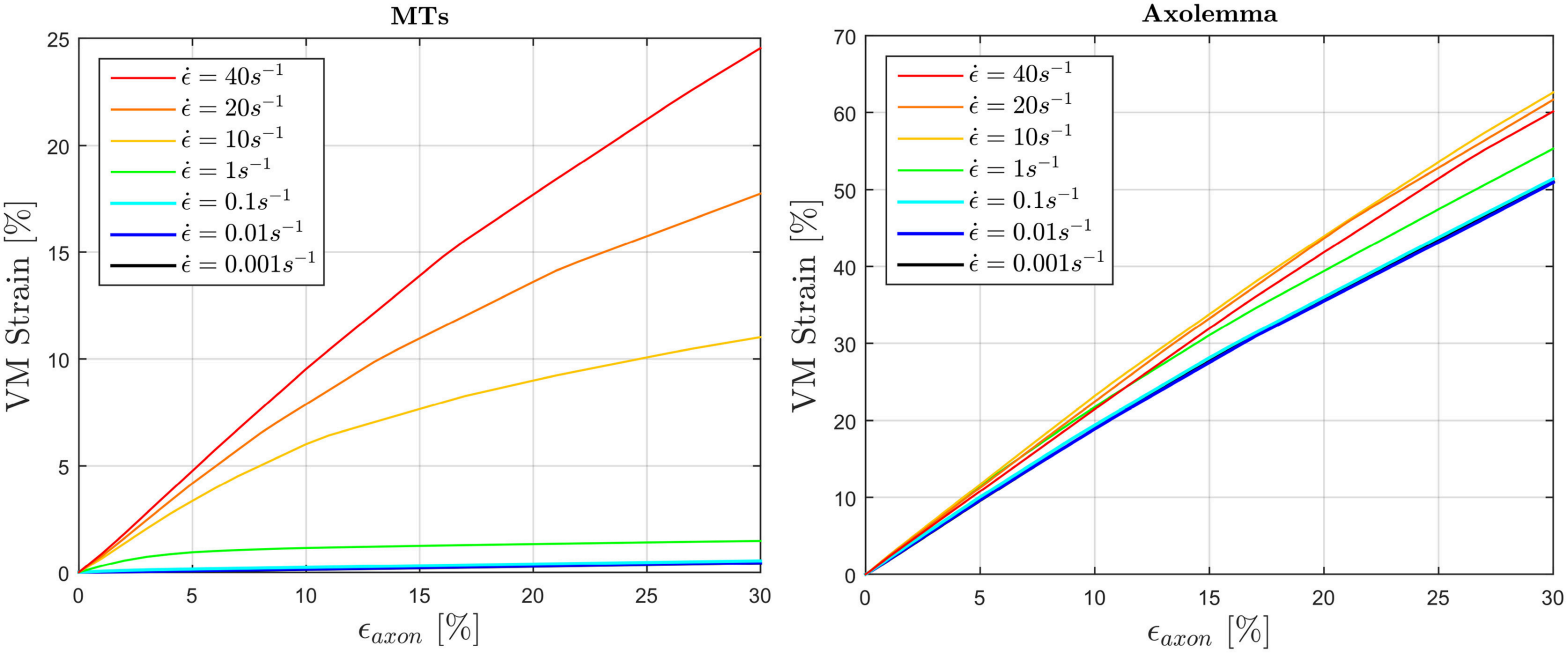
Average (over 10 models) maximum von Mises strain in the MTs bundle **(left)** and in the axolemma **(right)** computed when stretching the whole axon up to ε_*axon*_ = 30% at different strain rates ranging from the quasi-static to high dynamic regime.

**Figure 7:**
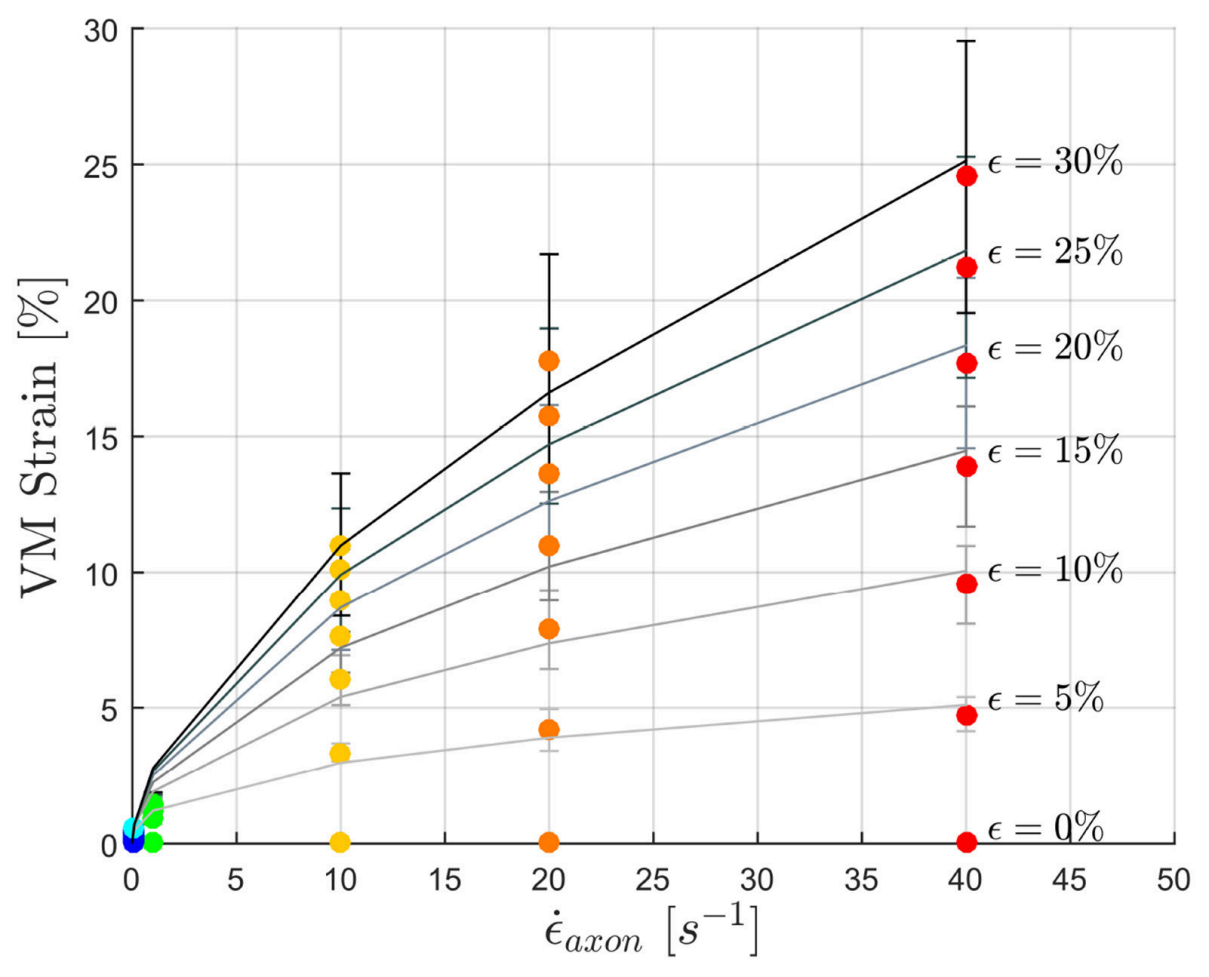
Maximum MTs strains as a function of applied strain rate. Color dots represent the data points. Gray-scale lines where produced by fitting the data with a power law with parameters in the following Table.

To fulfill the second aim of the study, high strain rate simulations were performed considering a simplified bundle kinematic. Figure 6 depicts maximum VM strains in MTs and axolemma compartment averaged over the family of 10 models. An increase in strain rate causes almost no increase in both MTs and axolemmal values when considering strain rates spanning the quasi-static interval (0.001−0.1*s*^−1^). However, as soon as the applied strain rate enters the dynamic regime, ϵ_*MTs, max*_ considerably increases. In particular, it is found that this quantity grows with the power of the strain rate. Figure 7 shows ϵ_*MTs, max*_ as a function of strain rate. Means over 10 data points (one for each of the 10 models for each strain and strain rate) are depicted as colored dots with error bars representing the data standard deviation, whereas greyscale lines represent the power-law fit ($\epsilon_{MTs,max}=a{\dot{\epsilon }}_{axon}^{b}$) at different strain magnitudes. Fitting parameters were derived with a nonlinear least squared regression in MATLAB and are reported in Table 2.

**Table 2:** Power Law fit parameters.

**$\epsilon_{MTs,max}=a{\overset{.}{\epsilon }}_{axon}^{\text{b}}$**						
ϵ	**5%**	**10%**	**15%**	**20%**	**25%**	**30%**
a	0.012	0.019	0.023	0.025	0.027	0.028
b	0.390	0.450	0.503	0.539	0.570	0.597

**Figure 8:**
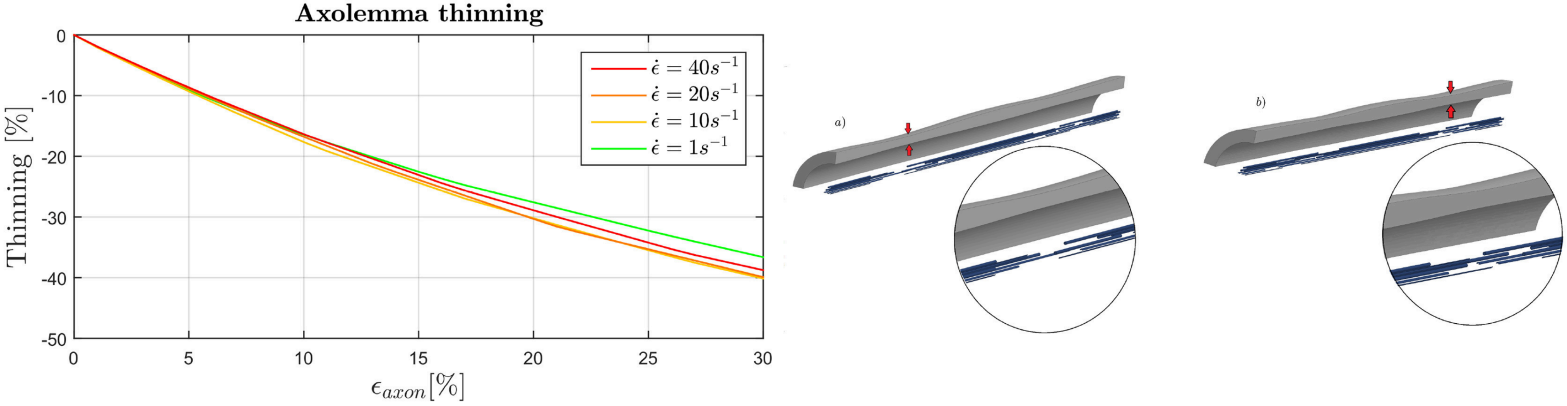
**(Left)** Evolution of the maximum thinning of the axolemma-actin complex (averaged over the family of 10 models) when the axon is stretched up to ϵ_*axon*_ = 30% in dependence of strain rate. **(Right)** Final configuration of model 4 (a) and 2 (b). Insets qualitatively show the correspondence of maximum thinning of the membrane and least MTs overlap.

In Figure 8 (left) the maximum thinning of the membrane-cortex complex is displayed as a function of the applied global strain. Results are shown only for the dynamic simulations as maximum thinning is an effect of interest as a potential injury metric for dynamics loading scenario. It can be noted that the elements of the axolemma complex that undergo maximum thinning experience a thickness reduction of the 40% when the axon is stretched up to a global strain of the 30%. With the increase of strain rate from $\dot{\epsilon }=1s^{-1}$ to $\dot{\epsilon }=10s^{-1}$ there is an additional 5% thickness decrease. This trend is inverted as the strain rate is further increased. Moreover, from visual inspection of the simulation results, maximum thinning occurs in correspondence of the minimum MTs overlap (or maximum MTs distancing), that is where the bundle becomes “slack” (Figure 8, right). In each of the simulations a weak spot such as the one here described appears within the 8μ*m*-long model.

### 3.3. Effect of tau protein failure on maximum compartmental strains

**Figure 9:**
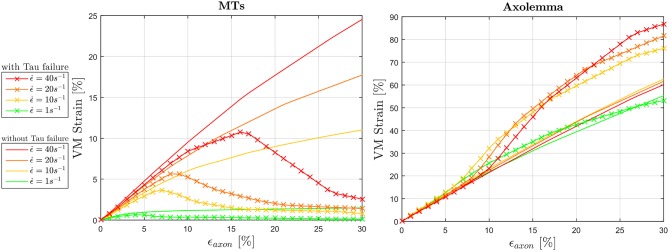
Effect of tau protein failure on maximum VM strains experienced by the MTs **(left)** and the axolemma **(right)** for dynamic strain rates ($\dot{\epsilon }=1,10,20,40s^{-1}$). VM strains computed in simulations where tau protein failure feature was included (solid line with crosses) are plotted against the same quantities computed in simulations that did not include this feature.

The effect of tau proteins failure on the mechanics of the composite axonal structure was also studied by including a strain failure threshold for these elements. Figure 9 illustrates the resulting ϵ_*MTs, max*_ and ϵ_*Axol, max*_ (averaged among 10 models) in comparison with those previously obtained with intact tau proteins. As a result of cross-links failure, the maximum strains sustained by the MT bundle drop. By failing progressively, these proteins exert less and less traction on the bundle filaments, until bundle disconnection. This disconnection is localized where previous simulations with intact tau proteins resulted in a “slack” bundle portion with minimum MTs overlap. A strain rate dependence of both the VM strain peak values and peak occurrences can be observed: the higher the strain rate, the higher the strain reached and the more delayed is the occurence of bundle failure by means of globally applied strain. Should we define bundle failure as the instant at which the maximum strain drops, then this takes place respectively at ϵ_*axon*_ = 4, 7, 8, and 16% for strain rates $\dot{\epsilon }=1$, 10, 20, and 40*s*^−1^. It is worth noting that, in our model, even when the MT bundle is fully disconnected, the strain along these filaments does not drop to zero, due to the remaining connection with the rest of the axonal filaments and membrane. While tau proteins failure reduces the maximum VM strains in the MTs, this has an opposite effect on the maximum VM strains in the axolemma. The interplay between these two compartments determines a final increase in axolemma VM strain, respectively of 25, 34, 43% for $\dot{\epsilon }=10,20,40s^{-1}$ with respect to the simulations with intact tau proteins. Introducing tau proteins failure also restores the expected trend in the axolemma maximum VM strain magnitudes with the increase of strain rate. In particular, while previous results (Figure 6, right) showed a trend inversion at $\dot{\epsilon }=40s^{-1}$, in Figure 9 (right) maximum VM strains reached at this strain rate are higher than those reached at $\dot{\epsilon }=20s^{-1}$. The same amount of difference is found also for membrane thinning and hence these results are not reported.

## 4. Discussion

In this study an axonal FE model—including all mechanically relevant substructures—was developed, validated and was used in large strain and high strain rate scenarios to analyze axonal injuries from a structural mechanics perspective. Results show that axonal components (MT bundle and axolemma) cannot be studied in isolation to infer axonal damage thresholds. Due to its heterogeneous nature and the interplay between different structures, the current results suggest that globally applied deformations (ϵ_*axon*_) do not directly translate to subcellular deformations. The model helps to overcome the ambiguities that exist in the field about the interpretation of experimentally observed phenomena. In particular, the model provides us with important local component level information that supports the hypothesis of axolemma mechanoporation as a candidate for axonal injury trigger.

Previous studies have hypothesized MTs failure as the cause behind axonal injury based on experimental observations (23, 24). Others have tried to computationally analyze this failure mechanism (28, 29, 31–33) comparing computationally-derived MTs strains against MTs failure thresholds sometimes idealizing the MT bundle kinematic. In an effort to assess the size effect of this idealization, it was shown that 3D kinematics and in particular MTs bending substantially contribute to maximum strains in the MT bundle. This error was quantified and found to be deterministic in our framework and could in turn be used as a multiplicative correction factor ($cf=\frac{1}{1-e}=2.33$) when assuming a simplified bundle kinematic. Taking into account this aspect seems essential, especially when using MTs failure [ϵ_*f*_ = 50% (73, 74)] as a term of comparison for a potential cell injury metric.

Although our results confirm the strain rate dependence and hence potential vulnerability of the MT bundle, they show that a more likely cell injury cause is to be found in the large deformations that the axolemma undergoes, at least in the strain and strain rate ranges considered in the present study. In particular, even when considering a correction factor *cf* for the computed maximum VM strains (Figure 6, left) at the highest strain rate, the MTs failure threshold is exceeded only around ϵ_*axon*_ = 25%, which is higher than most of the axonal damage thresholds currently proposed (68, 69, 75, 76). It could be argued that the resulting MTs strain is dependent on the mechanical properties assigned to the tau proteins. Nevertheless, the authors want to stress that a stiffer behavior of these cross-links would also lead to a higher—and hence less realistic—effective stiffness for the whole axon, that was instead shown to be in good accordance with experimental studies (Figure 3). Moreover, in light of the results obtained when considering tau protein failure, it can be said that yhe MTs failure threshold is never reached by our model in the current framework. This confirms results by Peter et al. (28) that observed cross-links failure and MT pullout rather than MT failure, although their simulations were performed at higher strain rates.

In the current study, the behavior of an axolemmal complex interconnected with the other axonal substructures was for the first time quantified. Quantities such as maximum strains and membrane thinning were considered to describe the mechanical status of this compartment. The plasma membrane has been the object of many studies striving to determine rupture thresholds either though experiments (77, 78) or numerical simulations (79–81). Rupture has been found to be dependent on lipid composition and applied strain rate. Pore formation for cholesterol-rich membranes [such as the axonal membrane was previously considered (36)] at high speeds was recently shown to fall between 100 and 200% areal strain (before scaling) with molecular dynamic simulations (80) and around 30% with experiments conducted on red blood cells (82, 83). However, our results cannot be put in direct comparison with these quantities because the deformation applied in these studies is of a biaxial kind, whereas our shell elements mainly undergo uniaxial deformation and lateral thinning. Moreover, it must be stressed that our shell elements represent the membrane-actin cortex complex rather than the sole plasma membrane. That being said, it is interesting to notice that in the axolemma model, 30% maximum strain is reached when ϵ_*axon*_≈15% (or ϵ_*axon*_≈10% when tau protein failure is enforced) at dynamic strain rates, values that are in accordance with previously proposed axonal injury thresholds (67–70). More importantly, in this study for the first time the localization of maximum strains in the axolemma was shown. This potential damage localization was found to be related to axolemmal maximum thinning and minimum MT overlap—or bundle disconnection when tau proteins were allowed to fail. Considering the axon as a periodic repetition of our 8μ*m*-long axonal FE model, this could explain the periodic accumulation of tau proteins that appears [at a distance <10μ*m* (23, 24)] on the axon as a consequence of an injurious load.

Although this study gives new insights about the axonal injury mechanism and proposes a new comprehensive approach to address the latter, it is not free from limitations. First and foremost, due to lack of univocal geometrical and mechanical characterization, the axonal inter-structural connections (between NFs and NFs, as well as NFs-axolemma) are not specifically defined in the current study. The modeled NFs compartment itself, as initially stated, does not closely replicate the real NF network. It is not excluded that in reality the mechanical properties of this network and its connections with the rest of the filaments would affect axonal behavior under dynamic loads such as proposed in Grevesse et al. (14). in particular, although it is currently supposed that cross-bridges do connect the NF network with axolemma and microtubules (55), should this connection be transient (due to failure of these connections or simple debonding) rather than fixed as the one presented in this model, the entire axonal structure would eventually loose its connectivity. As a result, the deformation of the MTs bundle would not affect the deformation of the membrane as much as it does now. Until the mechanical behavior of these links is uncovered by further research, it can be considered fair to assume the cytoskeleton as a fully connected structure.

Moreover, our model does not account for polymerization/ depolymerization of the MTs. A recent publication (33), however, shows that the effect of including MTs polymerization and depolymerization, though more pronounced at slow strain rate, is to be considered marginal at high rates. This is to be expected given that polymerization/depolymerization times are of ∽1, 000 ms, while our dynamic simulations have a duration of ∽10 ms. Furthermore, no other active molecular mechanisms such as NFs transport or crosslinks rebonding is taken into account in this study. All model components mechanical properties remain unaltered and linear throughout the simulations (with the exception of failing tau proteins). Tau detachment and reattachment in absence of any applied force is governed by thermal fluctuations. While necessary to study physiological behavior such as axonal elongation, it was considered of less importance in this study aimed at understanding the primary effect of dynamic injury loads.

Given that primary axotomy (immediate severing of the axon) is a non common event in DAI (84), it is crucial to identify what is the trigger of the series of secondary events in the evolution of axonal damage. Several molecular pathways, that lead to morphological changes (undulations and axonal swellings) and in turn lead to secondary axotomy have been proposed (84) and it is important to note that this *in-silico* model cannot capture them. What this structural approach aimed at, was the definition of an injury trigger. To capture the damage evolution following the mechanical insult it is indeed necessary to include not only more sophisticated and active properties but also molecular details. Future research should aim at understanding, for example, what could be the consequences of the axolemmal deformations that were reported here. Are these deformations sufficient to cause a direct mechanoporation of the lipid bilayer or the opening of protein channels, which has been proposed to cause influx of calcium and consequent calpain activation (84)? Moreover, when the load ceases to be applied, how do membrane lipids or the actin-spectrin cytoskeleton behave? These are all questions that a pure structural mechanics approach cannot answer and that need to be analyzed at a molecular scale.

Several studies so far have proposed advanced brain models that take into account structural information for the computation of axonal strains, specifically, strains in the direction of the white matter tracts) (7–9). However, these studies assume axons as homogeneous rod-like entities and evaluate the brain response using a posteriori-computed injury criteria. This study sheds light on the mechanism behind axonal injury from a mechanical perspective and lays the basis for future studies where more sophisticated as well as more axon-specific properties could be considered. New injury metrics could be investigated, leading to the definition of well-informed injury criteria that could be embedded at the tissue and organ level.

## 5. Conclusions

In this study, a comprehensive model of a representative volume of an axon was presented. The model consists of three main substructures: the membrane-actin cortex complex, the MT bundle and a discrete elements mesh generally representing the NFs network. This detailed model was shown to correlate well with forces measured in axonal stretching experiments. The results show that it is necessary to take into account a realistic MT bundle kinematic (either by modeling it explicitly or by using a correction factor) when computing maximum MT strains. In addition, based on the dynamic simulations results, axolemmal disruption is put forward as a cell-injury trigger. More specifically, a strain concentration area on the axolemma was systematically found to be localized where tau proteins extensively fail leaving the MT bundle disconnected. All in all, this study shows that, when trying to reveal the axonal injury mechanisms, the axon cannot be considered as a homogeneous material, neither can only isolated parts of it explain axonal injury mechanism. It is instead necessary to consider the axon in its fully connected multi-structural entirety.

## Author contributions

AM and SK contributed to study conception. AM designed the study, performed the analyses, and wrote the manuscript. SK critically reviewed and approved the manuscript.

### Conflict of interest statement

The authors declare that the research was conducted in the absence of any commercial or financial relationships that could be construed as a potential conflict of interest.
